# The Role of Cytokines and Chemokines in Filovirus Infection

**DOI:** 10.3390/v7102892

**Published:** 2015-10-23

**Authors:** Sandra L. Bixler, Arthur J. Goff

**Affiliations:** United States Army Medical Research Institute of Infectious Diseases, 1425 Porter St, Frederick, MD 21702, USA; arthur.j.goff.civ@mail.mil

**Keywords:** Filovirus, Ebola, Marburg, cytokine, chemokine, immunopathology

## Abstract

Ebola- and marburgviruses are highly pathogenic filoviruses and causative agents of viral hemorrhagic fever. Filovirus disease is characterized by a dysregulated immune response, severe organ damage, and coagulation abnormalities. This includes modulation of cytokines, signaling mediators that regulate various components of the immune system as well as other biological processes. Here we examine the role of cytokines in filovirus infection, with an emphasis on understanding how these molecules affect development of the antiviral immune response and influence pathology. These proteins may present targets for immune modulation by therapeutic agents and vaccines in an effort to boost the natural immune response to infection and/or reduce immunopathology.

## 1. Introduction

### 1.1. Filoviruses

Filoviruses are approximately 80 nm in diameter and are filamentous in shape. The 19 kb genome is composed of a single linear non-segmented negative-sense RNA that encodes 7 genes: nucleoprotein (NP), the polymerase cofactor VP35, the matrix proteins VP40 and VP24, glycoprotein (GP), the transcription activator VP30, and an RNA-dependent RNA polymerase (L). The family *Filoviridae*, which is composed of a group of enveloped, negative-sense ssRNA viruses, is divided into three genera: *Ebolavirus*, *Marburgvirus*, and *Cuevavirus* [1]. There are five ebolaviruses: Ebola virus (EBOV), Sudan virus (SUDV), Taï Forest virus (TAFV), Bundibugyo virus (BDBV), and Reston virus (RESTV) [1]. There is a single cuevavirus (*Lloviu virus*), as well as two marburgviruses, Marburg (MARV) and Ravn (RAVV) [1].

MARV was first identified in 1967 as the causative agent of simultaneous outbreaks of viral hemorrhagic fever in Marburg and Frankfurt, Germany and Belgrade, Yugoslavia [2]. The origin of the outbreaks was traced to laboratories that utilized African green monkeys and their tissues [3]. In 1976, large outbreaks of hemorrhagic fever with high mortality occurred in Sudan and Zaire (now the Democratic Republic of Congo (DRC)) [4,5,6]. Although MARV was initially suspected to be responsible for these outbreaks, further testing indicated the presence of related but previously unknown filoviruses designated EBOV and SUDV.

Of the five ebolaviruses, EBOV, SUDV, and BDBV have been associated with human outbreaks in Equatorial Africa, reaching lethalities of 30%–80% [7,8]. Only a single case of TAFV has been identified—in a researcher performing necropsies on infected chimpanzees in 1994 [9]. While that individual became clinically ill, they ultimately recovered [9]. Like TAFV, RESTV has not caused any lethality in humans; serological evidence from humans suggests that exposure to RESTV could lead to a subclinical infection [10,11]. RESTV is highly pathogenic and fatal in nonhuman primates and has been associated with large outbreaks in various primate colonies, including the United States [12,13].

The largest Ebola virus disease (EVD) outbreak in history is currently in progress in Western Africa [14,15]. Beginning in March 2014, widespread transmission of EBOV occurred in Liberia, Sierra Leone, and Guinea [14,15]. Due to the ease of modern international travel, a limited number of cases of EVD were exported to other countries throughout the world, including Spain, Germany, Italy, Great Britain, and the U.S. [14,15]. As of 29 September 2015, 28,388 total cases have been reported, with 11,296 deaths [14,15].

While humans are clearly capable of being infected with filoviruses, the high mortality rates suggest that they are not the natural reservoir. Virological and serological evidence obtained from the testing of numerous species of mammals, insects and other animals in Africa have suggested that bats are likely a natural reservoir [16,17,18]. Filoviruses can also be isolated from animals of other species, including pigs [19] and nonhuman primates (NHPs) [20]. As they accurately recapitulate the clinical and pathological picture of human filovirus infection, nonhuman primates serve as the gold standard animal model for studying filoviruses in the laboratory [21]. Although they are not lethally infected with wild-type filoviruses, repeated passage of filoviruses in animals can render these viruses capable of lethally infecting mice [22,23,24,25], hamsters [26], and guinea pigs [27,28,29], thus providing small animal models for studying pathogenesis and evaluating medical countermeasures.

### 1.2. Filovirus Disease

Filovirus transmission can occur through several different routes. As humans are not the natural reservoir, initial transmission most likely occurs through contact with an infected animal, with the virus gaining entry through mucosal surfaces or breaks in the skin [30,31,32]. Subsequent individuals can become infected through direct contact with EVD/Marburg virus disease (MVD) patients, contact with their bodily fluids, or through nosocomial transmission resulting from exposure to contaminated needles or other medical objects [33,34]. Most importantly, close contact is required for transmission, putting healthcare workers and family members caring for infected individuals at the highest risk for infection [33,34]. Interestingly though, transmission studies of the 1995 EVD outbreak in Kikwit, Zaire revealed that only 16% of household contacts of primary EVD patients became infected [34].

The incubation period of EVD and MVD is highly variable and can range from 2 to 21 days [34,35,36]. Early symptoms of filovirus infection are flu-like in nature and resemble the prodrome of many other viral infections [36,37]. These non-specific symptoms include headache, fever, myalgia, fatigue, and gastrointestinal symptoms such as diarrhea, vomiting, and abdominal pain [36,37]. As the disease progresses, a maculopapular rash may appear in some cases on days 5–7 [37,38]. Later stages include systemic symptoms, indicating the involvement of multiple organ systems and resulting in gastrointestinal, respiratory, vascular and neurological complications [36]. Death occurs between 6–16 days following the initial appearance of symptoms, and is often the result of multi-organ failure and shock [36,39]. Although EVD and MVD are characterized as hemorrhagic fevers, hemorrhagic symptoms only manifest in just under half of all patients and include conjunctival and GI bleeding, in addition to the maculopapular rash [36,37,38].

The clinical manifestations of filovirus infection are directly related to the extensive underlying pathology that occurs. Organ damage is widespread, particularly in the liver, spleen, and kidneys; therefore, common laboratory findings include elevated levels of liver enzymes such as alanine aminotransferase (ALT) and aspartate aminotransferase (AST) and potential markers of kidney damage such as blood urea nitrogen (BUN) and creatinine [3,37,40]. Disruption of the endothelium results in fluid distribution complications and an imbalance between the intravascular and extravascular spaces, leading to hypotension and shock.

Unsurprisingly, coagulation abnormalities are a hallmark of hemorrhagic fevers. Disruptions in hemostasis manifest in the form of reduced platelet counts (thrombocytopenia) and red cell destruction [37]. Coagulopathy, along with endothelial dysfunction, may be linked to organ damage and ultimately organ failure, as potential formation and deposition of clots within blood vessels can impede blood flow to these organs, resulting in necrosis [37]. As such, fibrin thrombi, ischemia, and multifocal necrosis are all common pathological findings in filovirus infection [40]. The formation of clots within vessels may also deplete the pool of available clotting factors; in combination with thrombocytopenia, this likely contributes to hemorrhagic manifestations of EVD and MVD [35].

Analysis of human samples and longitudinal NHP samples from animal studies has shown that EVD is associated with a strong pro-inflammatory cytokine response [41,42,43]. This “cytokine storm”, which has been observed in other infectious diseases including influenza, has been linked to a dysregulated immune response and higher mortality [41,43,44,45].

### 1.3. Cellular Tropism of Filovirus Infection

Initial targets of EBOV and MARV infection include cells of the immune system, such as monocytes and macrophages [24,27,46,47,48,49,50]. The migratory capacity of these cells likely aids in viral dissemination to multiple organ systems [51,52]. As infection progresses, EBOV and MARV demonstrate tropism for a wide variety of cell types [24,47,48,49,50,53,54,55], with the notable exception of lymphocytes, which do not appear to be productively infected with filoviruses [47,48,49,54,56]. As the infection of endothelial cells does not occur until the end of the disease process, the endothelial dysfunction observed is likely due to production of cytokine mediators, and not due to direct viral infection of those cells [36,47]. However, it has been suggested that GP shed from EBOV may directly modulate endothelial cell permeability [57]. *In vitro* infection of monocytes and macrophages leads to their activation and subsequent production of pro-inflammatory cytokines, which are highlighted in further detail below.

Unlike monocytes and macrophages, *in vitro* infection of dendritic cells (DCs) with filoviruses does not result in cytokine production [58,59]. Moreover, dendritic cells are impaired in their ability to upregulate costimulatory molecules and induce T cell activation in response to infection [58,59,60]. Additionally, filovirus infections are characterized by massive lymphocyte apoptosis, which may be due, in part, to antigen presenting cell dysfunction [24,25,41,46,47,49,54,61,62,63,64] and a poor resulting cellular immune response [41,62,65,66,67]. However, the impairment of dendritic cells may be cytokine-specific, as MARV-infected DCs still produced TNFα in response to lipopolysaccharide (LPS) stimulation [58].

## 2. Cytokine Families and Their Role in EVD/MVD

**Figure 1:**
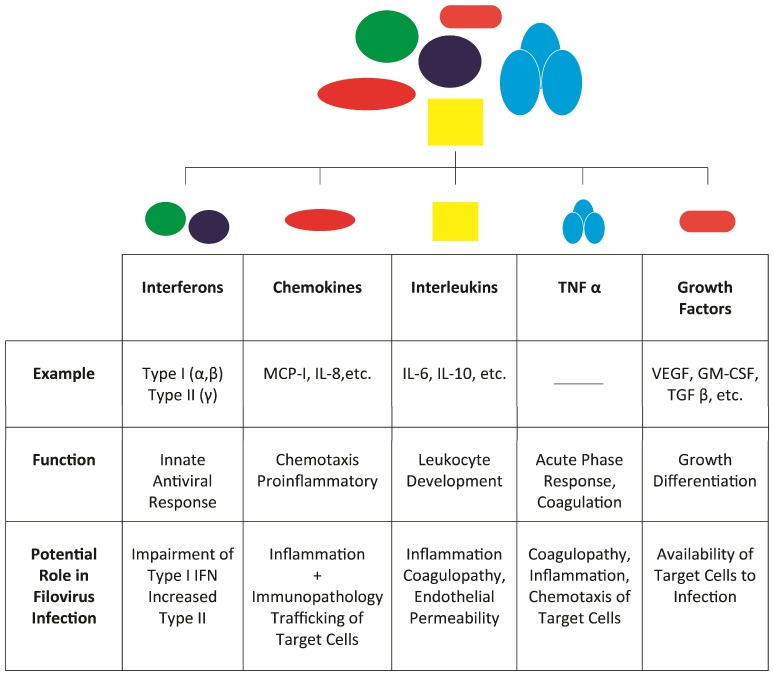
Cytokine families and their functions.

The term cytokine refers to a set of small proteins less than 20 kDa that regulate various biological and immune processes. Classes of cytokines have been traditionally defined by structure or function, with interleukins, growth factors, chemokines, and interferons constituting the larger cytokine family (Figure 1). In general, filovirus infection is characterized by high levels of pro-inflammatory cytokines and chemokines, the majority of which are likely produced by infected monocytes and macrophages. These include interleukin (IL)-1β, IL-8, IL-15, IL-18, macrophage inflammatory protein-1α (MIP-1α) and -β, monocyte chemoattractant protein-1 (MCP-1), interferon gamma inducible protein-10 (IP-10), gro-α, and eotaxin, among others. Interestingly, low levels of T cell-associated cytokines such as IL-2 are found in infection [53]. This may be explained by the massive lymphocyte apoptosis observed following EBOV and MARV infection. Below, we discuss in further detail the role of various individual cytokines in EVD and MVD.

### 2.1. Interferons

The type I interferons include interferon (IFN)β and several variants of IFNα. Type I interferons function as an early innate immune response to viral infection [68]. They are produced by virally infected cells in response to various signals, including pattern recognition receptors (PRRs) and toll-like receptors (TLRs) [68,69]. As their name suggests, type I interferons “interfere” with viral infection through the activation of gene transcription programs. Their first function is to serve as an early warning system for neighboring cells and induce an antiviral state in those cells to prevent additional infection [70]. Interferons activate the Janus-associated kinase (JAK)/signal transducer and activator of transcription (STAT) signaling pathway, which regulates expression of genes that prevent viral replication [70]. In doing so, interferons promote the activation of innate immune cells such as macrophages, dendritic cells, and natural killer (NK) cells, which aid in recognition of pathogens and virus killing [70]. STAT activation by type I interferons also promotes expression of major histocompatibility complex-I (MHCI), which is expressed on all nucleated cells and is a key component of antigen presentation to CD8+ T cells [70].

Filovirus infection interferes with the type I interferon response *in vitro*. EBOV VP35 inhibits production of type I interferons by blocking dsRNA-dependent protein kinase and the interferon transcription factors IRF3 and IRF7 [71,72,73,74]. EBOV VP24 inhibits the transcription factor STAT1 through direct binding, impeding nuclear translocation of STAT proteins and both types I and II interferon responses [75,76]. MARV VP35 also inhibits type I interferon responses [36,58]; although the exact mechanism has not been defined, sequence homology between EBOV and MARV VP35 suggests that the two may utilize a similar mechanism [77,78]. Like EBOV, MARV also inhibits type I IFN-induced tyrosine phosphorylation of STAT1 and STAT2; however, this is mediated by VP40 in the case of MARV and not VP24 as with EBOV [79,80]. MARV VP40 does so by inhibiting phosphorylation of Janus kinases, particularly JAK1 [79].

Animal modeling has demonstrated that type I interferon responses likely play an important role in the natural resistance of adult immunocompetent mice to fatal filovirus infection. While immunocompetent adult mice are not lethally infected with EBOV, SUDV, or MARV [22], repeated serial passage in young or immunodeficient mice generates mouse-adapted EBOV [23], MARV [25], and RAVV strains [81] that are capable of producing lethal infection in immunocompetent mice. Sequencing of the mouse-adapted RAVV revealed mutations in VP40, suggesting that type I interferon inhibition by the virus may be a key component of the resistance of healthy mice to infection with filoviruses [25,81,82]. In further support of this, non-adapted MARV, EBOV, and SUDV strains are able to infect IFN receptor knockout mice, and treatment of immunocompetent mice with antibodies against IFNα/β renders them susceptible to wild-type strains of EBOV [22].

Importantly, antagonism of interferon responses was associated with increased virulence in filovirus infection. Analysis of gene expression in liver cells infected with EBOV, RESTV, or MARV revealed that infection with the highly pathogenic EBOV and MARV strains resulted in global inhibition of innate antiviral signaling pathways, particularly the type I interferon response [80]. Interestingly, while EBOV and MARV inhibited the vast majority of interferon-stimulated genes (ISGs), a small subset of these genes was actually activated in RESTV infection, which is known to be less pathogenic in humans [80]. It is possible that activation of these immune genes may affect the virulence of RESTV in humans, resulting in lower morbidity and mortality.

Studies have proposed an additional antiviral role for type I interferons in filovirus infection. Treatment of 293T cells with IFNα was found to inhibit the release of pseudotyped Ebola virions from those cells [83]. The cellular factor that restricted viral release was later identified to be the protein tetherin, which is induced by type I interferons and also restricts particle egress in other viral infections such as HIV [84,85]. Later studies revealed that EBOV and MARV GP both counteracted the inhibitory effects of tetherin on the release of viral progeny from infected cells [86].

Based on the *in vitro* data concerning the antiviral effects of type I interferons on filoviruses, type I interferons are not likely to be highly induced in fatal infections. In support of this, expression of IFN-α was actually increased in patients who survived SUDV infection, suggesting that the ability to overcome the inhibitory effects of filovirus infection on type I interferon responses may play a role in disease outcome [87]. Moreover, treatment of NHPs with IFNα2b [88] or IFNβ [89] resulted in delayed viremia and time to death, although no effect on mortality was observed. Post-exposure vaccination with virus-like particles (VLPs) containing GP, NP, and VP40 induced type I IFN signaling in mice; these mice had reduced levels of inflammatory cytokines and chemokines and increased survival [90,91].

Although there is a fair amount of evidence to suggest that increased IFN may be protective during filovirus infection, data does exist which suggests the contrary. One study found elevated levels of IFNα in fatal human cases of EBOV [92]. Similarly, IFNα2 expression was higher during the acute phase of BDBV infection in patients who did not survive infection, as opposed to those who did [93]. However, *in vitro* infection of macrophages and PBMCs with EBOV did not induce type I interferon production in these cells, while *in vitro* infection of cells with BDBV only resulted in a small and transient increase in type I interferon early in infection (day 3 post infection (pi)) [94]. Finally, type I interferons (IFNα and -β) were elevated in sera from NHPs infected with EBOV [46,95]. The data for MARV is similarly inconclusive, as elevated levels of IFNα were observed in mice [25] and in one study in NHPs [48], but not a second study in NHPs [47].

The interferon family also includes the type II interferon IFNγ; although it shares the interferon designation and also aids in the antiviral immune response, it plays a distinct role from type I interferons. While most cells are capable of IFNα and -β production, production of IFNγ is mainly restricted to cells of the immune system, including T cells and NK cells among others [96,97]. Additionally, type II interferon plays a dual role both in innate immunity and in the adaptive response [96,98]. IFNγ production is a hallmark of a T helper (Th)1 response and induces upregulation of both MHCI and MHCII, thereby promoting the function of CD4+ and CD8+ T cell responses [96,98]. IFNγ can also be pro-apoptotic and inflammatory in nature, as it activates the microbicidal functions of neutrophils and macrophages, such as phagocytosis and oxidative burst [96,98,99].

In EBOV infection, VP24 interferes with IFNγ-induced gene expression through inhibition of nuclear accumulation of phosphorylated-STAT1 [76]. MARV also inhibits IFNγ-induced gene expression, but does so through an alternative mechanism from EBOV. In MARV infection, VP40 inhibits JAK and STAT phosphorylation, which is required for downstream expression of IFNγ-induced genes [79].

The role of IFNγ in filovirus infection is controversial. As elevated levels of IFNγ have been observed in MARV, EBOV, and BDBV infection of NHPs and humans [43,62,92,93,100], a potential detrimental role for IFNγ in filovirus infection has been suggested. In these fatal cases, early production of IFNγ was followed by a subsequent loss of T cell-related mRNA expression and DNA fragmentation in leukocytes in several patients [62]. While IFNγ is likely required for activation of the immune response, it is also pro-apoptotic. It is possible that high levels of IFNγ during filovirus infection may promote apoptosis of T cells [101]. Additionally, IFNγ disrupts the endothelium [96], which may contribute to endothelial barrier leakage and pathology in infection. Conversely, the activation of cytotoxic T cells and subsequent viral clearance in survivors is thought to be associated with IFNγ [62]. IFNγ is also thought to be linked to protection observed in vaccine studies [102,103] and was a component of Ebola-specific CD4+ and CD8+ T cell responses in EVD survivors [104]. While the majority of the CD8+ T cells were single-producers of IFNγ, a significant number of CD4+ T cells not only produced IFNγ, but also IL-2 and TNFα [104]. Interestingly, unlike EBOV, BDBV, and MARV infection, infection with SUDV did not result in increased expression of IFNγ, suggesting that filovirus species may differentially modulate type II interferon expression [87]. The contradictory data concerning the role of IFNγ in filovirus infection may be due in large part to the broad and diverse roles that IFNγ plays in the immune response.

### 2.2. Chemokines

Chemokines are small (8–14 kDa) cytokines that are so named for their primary function in chemotaxis and cell trafficking [105]. The four types of chemokines are C, CC, CXC, and CX3C, with the majority in the CC or CXC categories [105,106]. Many chemokines are produced in response to stimulation or in response to other cytokines and primarily attract immune cells, such as monocytes and neutrophils, to sites of infection and inflammation (Table 1).

**Table 1:** Chemokines found in filovirus infection and their targets.

Chemokine	Alternate Name	Cells Attracted
CCL2	MCP-1	Monocytes
CCL3	MIP-1α	Monocytes, T cells, B cells, and eosinophils
CCL4	MIP-1β	Activated cells of the immune system, including T cells, B cells, and monocytes
CCL5	RANTES	Monocytes and activated T cells
CCL11	eotaxin	Eosinophils
CXCL1	gro-α	Neutrophils
CXCL8	IL-8	Neutrophils, basophils, eosinophils, macrophages, and T cells
CXCL10	IP-10	Monocytes/macrophages, T cells, NK cells, and DCs

In general, these chemokines have been found to be upregulated in filovirus infection. In EBOV infection of NHPs, increased levels of MCP-1, IL-8, MIP-1α, and MIP-1β have been found in serum, plasma, and transcripts from PBMCs [46,95,100]. Similarly, increased IL-8, MCP-1, and IP-10 were found in EBOV patients, with fatal cases associated with high levels of IL-8, MCP-1, MIP-1α and -β, IP-10, gro-α, and eotaxin [41]. In MARV infection, upregulation of IL-8, MIP-1α and -β, and MCP-1 have been found in infected NHPs [43,47,107,108]. However, there is a lack of data on chemokine expression in human MARV infection. In human SUDV infection, increased levels of IP-10 and Regulated on Activation, Normal T Cell Expressed and Secreted (RANTES) were observed, while IL-8 and MIP-1β were higher in non-survivors as opposed to survivors [87].

Data from *in vitro* experiments, animal studies, and human outbreaks suggest that differences likely exist in chemokine expression between the various *Ebolavirus* species. A side-by-side comparison of chemokine production in human cells infected *in vitro* with either EBOV or BDBV revealed that BDBV-infected cells produced less (2–5 log10) MCP-1 and MIP-1α than cells infected with EBOV; this difference in expression was attributed to the lower rate of replication for BDBV [94]. However, elevated expression of MCP-1 was observed in the acute phase of human BDBV infection and to an even greater degree in the convalescent phase [93]. As inflammatory chemokines have been associated with increased mortality, it is possible that species-specific differences in their expression may at least partially contribute to the differences in pathogenicity observed between the strains.

Data from NHP studies and from human infections suggest that high levels of pro-inflammatory chemokines in filovirus infection are a key component of filovirus pathogenesis and are linked to mortality. One of the likely primary effects of these chemoattractants is trafficking of inflammatory cells, which may contribute to tissue damage and pathology [109]. As monocytes and macrophages serve as cellular reservoirs for EBOV and MARV, a secondary effect may also be the migration of additional cell targets for the virus. Individually, these chemokines may have other roles in filovirus pathogenesis. For instance, elevated levels of IL-8 have been shown to antagonize the antiviral activity of type I IFN in other infections [110]. Along with direct inhibition of type I IFN by filovirus proteins, high IL-8 may contribute to inhibition of innate antiviral responses to infection.

### 2.3. Tumor Necrosis Factor

The prototypic member of the tumor necrosis factor (TNF) superfamily TNFα is produced by activated macrophages and other immune cells in response to inflammatory stimuli such as bacterial pathogen-associated molecular patterns (PAMPs) and IL-1 [111]. Accordingly, it plays a significant role in the inflammatory process, as it induces fever, attracts neutrophils, and stimulates phagocytosis by macrophages [111]. TNFα also serves as a component of the acute phase response in the liver, which leads to secretion of a variety of different factors including those involved in coagulation [111]. As such, it is a direct stimulant of the coagulation system. TNFα also activates vascular endothelial cells to express adhesion molecules such as selectins, intracellular adhesion molecule (ICAM), and vascular cell adhesion molecule (VCAM), thereby promoting migration of immune cells [111]. Finally, TNFα is capable of inducing apoptosis through activation of caspases [111].

In keeping with the pro-inflammatory environment induced by filovirus infection, elevated levels of TNFα have been detected in EBOV and MARV infection of NHPs [46,100,108,112], guinea pigs [113], and mice [25]. Increased expression of TNFα was also observed in human cells infected with BDBV and was comparable to what was observed for EBOV infection [94]. Interestingly, data collected during the acute and convalescent phases of human BDBV infection found that inflammatory cytokines such as TNFα were expressed at lower levels during acute infection as opposed to the convalescent phase [93], suggesting temporal differences in cytokine expression. Unlike EBOV and BDBV infection, elevated TNFα expression was not found in infection with SUDV [87]. It has been suggested that lower expression of TNFα, along with IFNγ, in SUDV infection may contribute to the longer time to death observed in SUDV infection as compared to EBOV [87].

Clinical manifestations of filovirus infection, such as fever, shock, and coagulopathy, may be in part linked to induction of TNFα during infection [114]. TNFα activates endothelial cells to express adhesion molecules, which promote the chemotaxis and extravasation of immune cells [111]. In filovirus infection, this may lead to an influx of antigen-presenting cells, which can serve as additional viral targets thereby enhancing infection. TNFα may also disrupt the endothelial barrier, leading to endothelial cell leakage [115,116]. In support of this, *in vitro* experiments have shown that induction of TNFα likely results in increased endothelial permeability [115,116]. Finally, the pro-apoptotic functions of TNFα may contribute to the apoptosis of lymphocytes observed in filovirus infection [49,115,117,118].

The combined detrimental effects of TNFα may be linked to mortality, as data from human patients suggests a correlation between high levels of TNFα and fatal EBOV infections [92]. Additional data from animal studies have suggested that the TNFα blockade during filovirus infection may be beneficial to survival. Treatment of MARV-infected guinea pigs with either TNFα antiserum [118] or an anti-TNFα antibody [113] resulted in increased survival, suggesting that inhibition of inflammatory cytokines may present an opportunity for therapeutic intervention during filovirus infection.

### 2.4. Interleukins

The designation interleukin was originally coined because these cytokines were believed to be produced solely by leukocytes [119]. Additional research has shown that while CD4+ lymphocytes are a source of some of these cytokines, a wide variety of other cell types are also capable of their production. Interleukins have been typically classified according to their specific role in the immune system—pro-inflammatory v anti-inflammatory, or Th1, Th2, Th17, *etc.* depending on the type of CD4+ response with which they are associated [120].

In addition to the massive production of pro-inflammatory cytokines, one of the hallmarks of filovirus infection is the impairment of lymphocyte responses as infection with EBOV or MARV induces lymphocyte apoptosis [24,25,41,46,47,49,54,61,62,63,64]. Historically, low levels of T cell cytokines (IL-2, IL-3, IL-4, IL-5, IL-9, IL-13) have been observed in fatal human cases of infection with EBOV [41] and in infection of NHPs with MARV [43]. In asymptomatic patients, increased T cell cytokine (IL-2, IL-4) transcripts in PBMCs were observed, indicative of T cell activation [121]. Moreover, IFNγ expression was also elevated, suggesting that cytotoxic T cells were likely activated and may have contributed to enhanced viral clearance [121]. In vaccine studies, protective responses against EBOV were associated with a Th1 memory phenotype, demonstrating the importance (and challenge) of generating long-lived T cell responses to filoviruses [103].

Although some discrepancies exist between individual studies, one common thread in filovirus infections appears to be elevated expression of IL-6. Increased expression of IL-6 was found in both plasma and spleen of MARV-infected cynomolgus macaques [48]; similarly, IL-6 was upregulated at the protein and transcriptomic levels in rhesus macaques infected with MARV [107,108]. Infection of adherent monocytes with EBOV *in vitro* resulted in secretion of IL-1β and IL-6, along with the chemokines IL-8 and RANTES [58]. Levels of IL-6 were elevated *in vivo* as well, as NHPs infected with EBOV showed increased expression of IL-6 in serum and plasma [46,95,100]. Moreover, high levels of IL-6 were associated with human EBOV [41] and SUDV fatalities [87].

In addition to high levels of IL-6, enhanced production of IL-1β also appears to be a commonality shared with other filoviruses. High levels of IL-1β are a feature of SUDV infection in humans [87] and were linked to fatal cases of EBOV [41]. Although elevated expression of IL-1β was observed with *in vitro* EBOV infection, IL-1β was not similarly induced following *in vitro* infection with BDBV [94]. Moreover, expression of IL-1β was significantly decreased during the acute phase of human BDBV disease as compared to the convalescent stage [93].

Increased expression of the pro-inflammatory cytokines IL-6 and IL-1β during filovirus infection is likely an important contributor to the clinical features of disease. These two cytokines have several overlapping functions; both serve as part of the acute phase response in the liver and are direct stimulants of the coagulation system [120]. As such, they may play an important role in coagulopathy which characterizes filovirus infection. Additionally, IL-6 serves as a transition cytokine, bridging the gap between the innate and adaptive immune response and functioning in monocyte recruitment and activation and differentiation of T cells [120]. IL-6 also may indirectly enhance infection and viral dissemination through recruitment of monocytes, which serve as viral targets. IL-1β is synthesized as pro-IL-1β and subsequently cleaved into its active form by caspases of the inflammasome complex. It stimulates vasodilation and expression of adhesion molecules on endothelial cells, thereby leading to extravasation of cells [120]. During filovirus infection, these functions of IL-1β may contribute to increased endothelial cell permeability and vascular leakage.

Other interleukins of interest in filovirus infection include IL-15 and IL-18, which have been found to be elevated in EBOV- and MARV-infected NHPs [95,100,108], and IL-16, IL-2, and IL-10 which have been linked to fatal EBOV infections [41,92]. IL-18 likely adds to the pro-inflammatory environment, as it stimulates production of other inflammatory mediators such as IFNγ [122].

Increased expression of IL-10 may also be important for species-specific differences between filoviruses, as elevated IL-10 expression was observed later in *in vitro* infection with BDBV but not EBOV [94]. It has been suggested that the high levels of IL-10 in BDBV infection may be at least partially responsible for the lower expression of pro-inflammatory cytokines observed during acute BDBV [93]. There may be differences in *in vitro* versus *in vivo* expression of IL-10 as studies of human BDBV infection found that elevated levels of IL-10 were present during the acute stage of disease but not during the convalescent stage [93]. However, IL-10 expression during acute infection was higher in non-survivors than in survivors [93]. In SUDV infection, expression of IL-10 was higher in non-survivors than in survivors [87].

Interestingly, the primary function of IL-10 is as an anti-inflammatory mediator [120]. The increased expression of IL-10 in fatal filovirus infections may be a concerted effort to dampen the overwhelming pro-inflammatory response but may exacerbate the deficiencies observed in adaptive immune response to infection. Modulation of IL-10 expression during filovirus infection may serve as a means to modulate pathogenesis, as inhibition of IL-10 in EBOV infection of mice resulted in survival and decreased inflammatory cytokine expression [123].

While historical data has suggested that T cell-associated cytokines are not highly expressed during filovirus infection, recent MARV data indicates that expression of these mediators may be temporal in nature. In a study that examined expression of various cytokines during a MARV serial sacrifice study in rhesus macaques, Th1 and Th2 cytokines were differentially expressed at various stages of disease [108]. Th2 cytokines such as IL-5 and IL-10 were found to be increased during the early stages of infection (days 3–4 pi), while Th1 cytokines such as IL-6, IFNγ, and IL-18 did not increase until later in disease (days 6–9 pi) [108].

### 2.5. Growth Factors

Growth factors are a subset of cytokines that promote the growth, differentiation, and proliferation of cells. These include factors such as vascular endothelial growth factor (VEGF) and platelet-derived growth factor (PDGF), which stimulate angiogenesis, and as well as colony-stimulating factors (CSFs), secreted glycoproteins that promote growth and differentiation of specific types of immune cells.

VEGF and PDGF both promote formation of blood vessels [124,125]. *In vitro* infection of hepatocytes with EBOV results in increased production of VEGF as early as one hour post-infection [126]. As VEGF is known to increase vascular permeability [124], it is possible that production of this mediator from infected cells may contribute to pathology observed in infection. However, it is not clear if VEGF is upregulated *in vivo* during filovirus infection. Several studies that examined a variety of cytokines during EBOV infection did not find any difference in expression of VEGF in EBOV-infected patients or NHPs as compared to controls [41,100,127]. Another study found that disabling of innate response antagonist domains within the EBOV protein VP35 resulted in increased cytokine expression, including PDGF and VEGF, suggesting that EBOV may negatively modulate their expression [60].

The three primary colony-stimulating factors are designated by the cell type they induce: macrophage colony-stimulating factor (M-CSF), granulocyte colony-stimulating factor (G-CSF), and granulocyte macrophage colony-stimulating factor (GM-CSF). M-CSF stimulates differentiation of hematopoietic stem cell precursors into macrophages, as well as phagocytosis [128]. GM-CSF promotes the production of monocytes, neutrophils, basophils, and eosinophils from stem cells and their subsequent release from bone marrow into systemic circulation [128]. G-CSF is produced by a variety of tissues and cells and stimulates production and release of granulocytes, particularly neutrophils, from bone marrow into circulation [128]. In addition to their roles as cellular growth factors, GM-CSF and M-CSF are now recognized as having pro-inflammatory properties [128].

Elevated levels of M-CSF and GM-CSF have been observed in EVD patients [41,125] and MARV-infected NHPs [108], respectively. More specifically, M-CSF is significantly elevated in fatal cases [41] of EBOV and SUDV infections with hemorrhagic manifestations, particularly in the later stages of disease [127]. In addition to its potential classical role in promoting neutrophil and monocyte release into circulation, GM-CSF in EBOV infection may play a direct role in modulating infection. Treatment of cells with GM-CSF led to increased permissiveness of those cells to EBOV infection *in vitro* [129]. Macrophages and dendritic cells demonstrated more efficient binding, internalization, and viral replication following treatment, suggesting that GM-CSF may modulate the cellular expression of the putative receptor(s) of EBOV [129].

Transforming growth factors were originally identified by their oncogenic transformation potential. Unlike the colony-stimulating factors, transforming growth factor-β (TGFβ) plays an important role in immune regulation and is known for its anti-inflammatory properties [120]. Interestingly, TGFβ is not only a key component required for differentiation of the anti-inflammatory Tregulatory (Treg) subset, but also of the inflammatory Th17 cell subset [120]. Enhanced TGFβ production has been observed from EBOV-infected hepatocytes *in vitro* [126]; it is not known what, if any, role this elevated expression may play in EBOV infection *in vivo*.

## 3. Unanswered Questions

Unanswered questions about the role of cytokines in viral hemorrhagic fever still remain. Discrepancies exist over the expression and roles of IFNα, IFNγ, and TNFα in EVD and MVD. These discrepancies may partially be explained by differences in the timing of the samples analyzed or differences in strains or variants of virus in question. Additionally, as filoviruses block both the production and signaling of interferons, it is likely that high levels of these molecules may be biologically irrelevant if downstream signaling and effector functions are blocked. Analysis of longitudinal samples obtained from a natural history study may be more informative about the temporal relationship of these cytokines during the course of infection and disease. Moreover, there is little known about the role that T helper cytokines such as IL-17, TGFβ and IL-10 may play. These cytokines are products of the inflammatory Th17 and anti-inflammatory Treg subsets, respectively [120]. These two subsets are differentially regulated and likely exist in a homeostatic balance [130]. Little is known about the regulation of these cells in EBOV and MARV infection and the potential role they may play in pathogenesis and the immune response to infection.

Another significant limitation in the filovirus field is that the cytokine data set is largely incomplete. The majority of these studies have been conducted by measuring systemic cytokine levels in serum, plasma, or transcripts from PBMCs. While this is likely due to the ease of obtaining samples at multiple time points throughout the course of disease, it vastly limits the scope of knowledge of cytokine expression during infection. A more detailed analysis of cytokine levels in various tissue compartments during infection would provide a more complete picture of filovirus immunopathogenesis.

## 4. Future Directions

Cytokines may provide potential treatment targets for filovirus infection, as modulation of immune responses may be used to improve patient outcome. One mechanism of achieving this is through regulation of the pro-inflammatory cytokine pathways that are associated with immunopathology and poor prognosis. This can be achieved directly by targeting these cytokines and/or cellular sources of those cytokines, or indirectly by enhancing anti-inflammatory cytokine production to restore immune homeostasis. By reducing these pro-inflammatory mediators, some of the clinical manifestations and cell and tissue damage may be reduced.

Some of these schemes are already been tested with varying levels of success. Chemokines have been an attractive target in drug trials for treating inflammatory disorders. Treatment of MARV-infected guinea pigs with anti-TNFα antibodies led to increased survival [113], suggesting that targeting of specific cytokines in filovirus infection may be an attractive route for therapeutic intervention. Indirect targeting of these inflammatory pathways may also be an alternative option. Treatment of EBOV-infected mice with mannose binding lectin (MBL) led to a decrease in pro-inflammatory cytokines (IL-1β and IL-17) and Th2 cytokines (IL-5,-10,-13) [131]. This was mediated by direct binding of MBL to the virus [131].

Instead of preventing inflammatory cytokine release during infection, cytokines may be used to improve the natural immune response to filovirus infection. Protection and survival have been associated with a combination of antibody responses, a strong cell-mediated immune response, and activation of cytotoxic T cells leading to viral clearance [62,103,132,133]. Specifically, cytokines may be used to stimulate T cell responses in an effort to restore homeostasis between Th1, Th2, Th17, and Treg populations and activate cytotoxic T cells.

Cytokines may also prove to be useful biomarkers for filovirus disease in predicting disease outcome for survivors and non-survivors. In general, a fatal outcome in filovirus infection has been associated with increased levels of pro-inflammatory cytokines and chemokines [41,43,45]. However, there are some discrepancies concerning the specific cytokines associated with mortality or survival. In keeping with the proposed negative role for inflammatory cytokines in filovirus infection, IL-6 and TNFα have been linked to fatal cases of EVD [41,87,92]. Interestingly, high levels of IP-10 and the anti-inflammatory cytokine IL-10 have been associated with fatal cases of EBOV in pediatric patients [134]. IL-10 may be required in small regulated concentrations in order to counteract the massive influx of pro-inflammatory cytokines. In cases of cytokine storm, a brief period of immunoparalysis typically follows the pro-inflammatory phase [44]. It is possible that continued release of elevated IL-10 prevents the required recovery from immunoparalysis. Alternatively, it has been suggested that lower expression of IL-10 early in BDBV infection may allow for an antiviral immune response to develop [94].

Alternatively, RANTES expression was associated with increased survival in pediatric EBOV cases [134]. As RANTES is known to function as a chemoattractant for activated T cells [135], improved adaptive responses in these patients may contribute to their survival. Additionally, elevated IL-6 and the early presence of IL-1β have been linked to survival in EBOV patients [42]. Although these results may initially appear to contradict the accepted doctrine concerning elevated cytokine expression, many of these pro-inflammatory cytokines are likely required early in filovirus infection to jumpstart the immune response to the virus. However, continued increased expression of these mediators as the disease progresses may ultimately lead to tissue damage and pathology.

**Figure 2:**
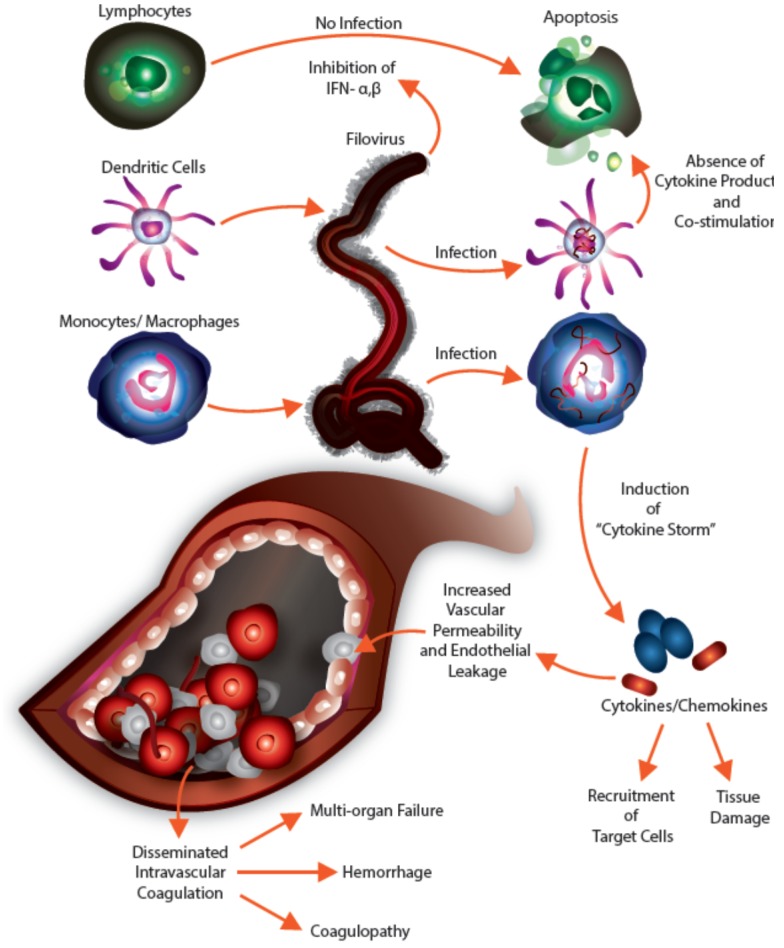
The role of cytokines in filovirus infection and immunopathogenesis.

Massive production of pro-inflammatory mediators during EVD and MVD is likely the result of infection of monocytes and macrophages, which serve as important sources of these molecules. While these cytokines are a necessary component of the innate immune response to viral infection, their unchecked production undoubtedly plays a significant role in the clinical and pathological features of EVD and MVD (Figure 2) and may present an opportunity for therapeutic intervention.
